# Editorial: Pathophysiology and therapeutic strategies for oral and head and neck cancers

**DOI:** 10.3389/froh.2025.1693338

**Published:** 2025-09-17

**Authors:** Vui King Vincent-Chong, Lee Peng Karen-Ng, Shamshad Alam

**Affiliations:** 1 Department of Oral Oncology, Roswell Park Comprehensive Cancer Center, Buffalo, NY, United States; 2 Oral Cancer Research and Coordinating Centre (OCRCC), Faculty of Dentistry, Universiti Malaya, Kuala Lumpur, Malaysia; 3 Department of Pharmacology and Therapeutics, Roswell Park Comprehensive Cancer Center, Buffalo, NY, United States

**Keywords:** oral cancer, head and neck cancer, therapeutic strategies, immunotherapy, prognosis, HNSCC, OSCC, chemotherapy

Head and neck squamous cell carcinomas (HNSCCs) contribute significantly to global cancer burden due to the advanced stage at presentation, high aggressiveness, and continuous poor survival outcomes (1). Although decades of research have uncovered a variety of genetic and molecular drivers of these malignancies, substantial challenges remain in the development of new therapeutics, and many aspects of their pathology and biology remain a mystery (1).

To improve our understanding of the complicated biology underlying head and neck cancer, this special issue on Pathophysiology and Therapeutic Strategies for Oral and Head and Neck Cancers was conceived. We seek to highlight innovative diagnostic approaches, novel treatment modalities such as targeted agents and immunotherapies, key molecular pathways, and novel biomarkers. Through this work, we hope to improve patient stratification, enable earlier detection, and improve outcomes for the millions of patients worldwide fighting for survival against HNSCCs.

Here, we present a multidisciplinary collection of 16 articles aimed at addressing various aspects of head and neck cancers. From biological mechanisms to innovative diagnostics, novel therapeutics, computational models, and rare clinical presentations, we hope to fully capture the mechanisms driving head and neck cancer behavior and the tools we have available to address it. We hope that this multifaceted collection will inspire further research and emphasize the importance of all disciplines of oncology, from the scientist's bench to the patient's bedside. Through collaboration, innovation, and inspiration, we hope that this Special Issue will further support scientific efforts to save lives and ease the suffering of patients with this persistent and destructive disease. Several original research articles in this collection focus on tumor biology and cellular behavior. Notably, Brach et al. demonstrated that high hydrostatic pressure (HHP), an approach that can be used to inactivate tumor tissue *ex vivo* for reconstructive surgery or tumor vaccine development, induces a dose-dependent cytokine release from HNSCC cells. The conditioned media from HNSCC cell post treatment of 200 MPa was found to enhance proliferation, migration, and invasion of HNSCC cells whereas 300 MPa led to devitalization and necrosis with an increased release of pro-inflammatory cytokines. Their findings highlight the dual role of HHP as both a potential devitalization approach and something that can promote tumor progression in HNSCC setting. Another study by Warner et al. indicated that a small population (range 3%–8%) of cancer stem cells (CSCs) in salivary gland adenoid cystic carcinoma with an expression of ALDH^high^CD44^high^ formed more spheroids and expressed higher levels of stem cell markers (Notch2, Bmi-1) *in vitro* and formed more lung metastasis *in vivo* compared to control ALDH^low^CD44^low^ cells, suggesting targeting this small fraction of CSCs is warranted using targeted therapeutic strategy. On the other hand, Khromov et al. explored the molecular crosstalk between mast cells (MCs) and oral squamous cell carcinoma (OSCC) cells in co-culture. Their analysis revealed activation of shared signaling pathways in both cell types and identified CCR2 and a subset of miRNAs correlated with prognosis in HNSCC patients. These findings suggest that MC-OSCC interactions converge on common inflammatory and pro-survival pathways and may represent novel therapeutic targets within the tumor microenvironment.

Among the collection, several studies have focused on improving treatment selection and tailoring strategies for HNSCC. For example, Lee et al. conducted a retrospective real-world analysis of KN048 and demonstrated that recurrent/metastatic (R/M) HNSCC patients who were HPV negative with a CPS score of 1–9 benefited more in terms of overall survival from combined chemotherapy and anti PD-1 therapy compared to anti PD-1 monotherapy, highlighting the incorporation of adjuvant chemotherapy with immune checkpoint blockades to enhance treatment efficacy; this warrants future clinical evaluation. In a complementary effort, Li et al. analyzed 1,539 stage II nasopharyngeal carcinoma (NPC) patients and showed that chemotherapy significantly improved cancer-specific survival in non-Asian patients with T1-2N1M0 stage but not in Asian patients. Kaplan–Meier analysis further demonstrated a poorer survival in non-Asian patients treated with radiation alone compared to those who received combined chemoradiotherapy. However, this observation was attenuated in an Asian population, highlighting that ethnicity and tumor stage can shape the benefits of combined modality treatment. Bridging these findings into the broader head and neck cancer spectrum for treatment optimization, Lv et al. have studied the standard of care for Kimura disease (KD), a rare chronic inflammatory disorder that mimics malignancy. By comparing surgical treatment with radiation therapy, they reported that radiation therapy yields superior local control compared to surgery alone, which was associated with higher recurrence rates. Wang and Wang conducted a systematic review and meta-analysis to clarify the prognostic impact of age and gender in papillary thyroid microcarcinoma (PTMC) managed with active surveillance. Their results show that patients who range from 30 to 50 years of age have a lower risk of disease progression, and gender was not associated with prognosis. These findings provide strong evidence to support individualized, risk-adapted management approaches for low-risk PTMC, reinforcing the importance of personalizing surveillance and intervention decisions to patient characteristics. Taken together with outcomes from other published studies in this field, these results show it is critical to optimize treatment strategies across the head and neck cancer spectrum.

Among the collection, there is a total of two comprehensive review articles that focus on tumor resistance and therapeutic response. Qin et al. provide a review highlighting how dysregulation of immunometabolism leads to therapeutic resistance in HNSCC and discuss how targeting key metabolic pathways that drive immune evasion may help overcome treatment resistance and provide new avenues for HNSCC immunotherapy. Another review by de Lima-Souza et al., whose group focuses on the Eph/Ephrin signaling axis, summarized its emerging role in OSCC and salivary gland cancers. Eph/Ephrin signaling has been implicated in tumorigenesis and therapeutic resistance. However, the precise molecular mechanisms underlying this tumor progression remain elusive. Their review article addresses these gaps by integrating recent molecular, clinical, and therapeutic studies and highlights the potential of Eph/Ephrin-directed approaches to support personalized treatment approaches in OSCC.

The collection from this special issue also consists of several studies that employed different approaches to improve precision diagnostics and prognostic modeling for head and neck cancer. For HNSCC, Li et al. developed an AI-driven pathomics model to predict the risk of oligometastasis in NPC, which is groundbreaking given that early identification of oligometastatic disease remains a major clinical challenge in NPC. Another study by Li et al. demonstrated that contrast-enhanced ultrasound (CEUS) assisted core needle biopsy significantly improves the diagnostic outcome for cervical lymph node tuberculosis, helping to avoid overtreatment in clinical practice, since cervical tuberculous lymphadenitis can be misdiagnosed as nodal metastasis of OSCC and lead to unnecessary functional surgery. For a subtype of head and neck benign lesions, Gao et al. investigated different markers to improve the diagnostic inaccuracies in giant cell rich lesions of the oral and maxillofacial areas. They showed that p63 immunohistochemistry strongly stains mononuclear stromal cells in giant cell tumors (GCTs) but not in giant cell reparative granuloma (GCRG). In addition, the characteristic “fluid–fluid level” on MRI serves as a helpful feature for diagnosing aneurysmal bone cyst (ABC), while tenosynovial giant cell tumors (TGCTs) are distinguished histologically by synovial monocytes, multinucleated giant cells, foam cells, and hemosiderin-laden macrophages. Overall, their study suggests that integrating IHC with radiologic hallmarks may improve diagnostic accuracy for this disease.

In this special issue, we also received four case reports that provided unique clinical insights that related to head and neck cancer. For instance, Yin and Gui contributed a case report of a rare subglottic adenoid cystic carcinoma (ACC), describing a patient who remained disease-free 15 years post initial diagnosis and treatment. In the case report, the authors highlight the potential for long-term survival in this uncommon laryngeal ACC subtype and provide valuable guidance for the diagnosis, surgical management, and follow-up of similar cases for future purposes. Zhan and Sun presented a rare case of extranodal NK/T-cell lymphoma of the nasal type and highlighted the diagnostic difficulty of this aggressive malignancy. As the clinical presentation can be non-specific, the authors indicated that early and accurate pathological diagnosis by combining cytology, histology, and immunophenotyping using markers such as CD56, granzyme B, and EBER improve patient prognosis. They also highlighted that the patient in this case had a longer disease duration than typically reported and yet still yields a good therapeutic response, emphasizing the importance of a comprehensive diagnostic plan and multidisciplinary collaboration in managing these aggressive rare lymphomas. Another aggressive and rare malignancy, undifferentiated pleomorphic sarcoma (UPS) of the sinonasal region, is presented by Wu et al. The authors report on a patient who progressed after surgery and chemotherapy but achieved a rapid and durable partial response following combination therapy with toripalimab (240 mg, Q3W) and anlotinib (orally once daily at 10 mg on days 1–14, followed by 1 week off, every 3 weeks per cycle). An immunohistochemical study demonstrated a high-grade undifferentiated phenotype, with loss of lineage-specific markers and over expression of Ki67, while expression of Fh, INI-1, H3K27me3, and SMARCA4 was retained. This case provides therapeutic confidence in the treatment of sinonasal UPS and that implementing the combination of toripalimab and anlotinib in future clinical trial is warranted. Another rare and clinically informative case report from Liu et al. describes tongue metastasis as the initial manifestation of clear cell renal cell carcinoma (ccRCC). They reported on a 62-year-old man who developed a painful mass on the anterior tongue, which histology and immunohistochemical analysis (vimentin+, CD10+, PAX-8+, EMA+) confirmed the diagnosis as metastatic ccRCC. Despite advanced treatment such as cytoreductive nephrectomy, metastasectomy, and following targeted therapy, the patient succumbed to the malignancy due to systemic infection. This unusual metastatic presentation in the oral cavity from the ccRCC highlights the need for more monitoring and effective treatments to improve the prognosis of this rare case in the future*.*

Together, these 16 contributions provide a panoramic view of current efforts to decode the biology and improve the clinical management of oral and head and neck cancers. From cutting-edge computational models to in-depth molecular analyses and rare clinical presentations, this collection underscores the importance of integrating basic, translational, and clinical research. We hope this Topic inspires further investigation, collaboration, and innovation in the fight against these complex diseases. We offer our sincerest thanks to all our authors, contributors, and reviewers for their hard work, time, and dedication to this special issue.
